# Reports of Two Cases of Taenia Saginata

**Published:** 1910-12

**Authors:** H. D. Eaton

**Affiliations:** Chihuahua, Mexico


For Texas Medical Journal.

### Reports of Two Cases of Taenia Saginata.

BY H. D. EATON, M. D., CHIHUAHUA, MEXICO.

I was much interested in reading Dr. Pfender's excellent article on tape worm in the February number of your journal. It is the first paper on the subject that I remember to have seen in any medical journal since I began practicing six years ago, and I found it very practical and helpful, for there are a great many sufferers from tape worm in this country (Old Mexico). It is impossible to get any accurate statistics on the subject, but making a rough estimate, I should say about one person in ten has an intestinal parasite of some kind.

Dr. Pfender makes the statement that the Taenia Saginata is by far the most common variety in the United States of America, only one authenticated case of Taenia Solium (or hog tape worm) having been reported, and I wish to report two cases of Taenia Saginata to help confirm Dr. Pfender's statement so far as Northern Mexico is concerned. It would be a good thing if every doctor who has had any experience with tape worms would report what he has observed, particularly as to symptomatology, because of its indefiniteness as known hitherto; and a disease which affects the health and vitality of such a large proportion of the population deserves to be studied more thoroughly in order to enable the average practitioner to suspect the presence of intestinal parasites and restore a large number of patients to health and to confidence in the profession who otherwise lose faith because of ineffectual and long drawn out treatment for "indigestion" or "nervousness" and who finally drift into the hands of some quack who gives them quick relief by removing the offending parasite.

Case 1. An American girl, age 6, lived all her life in Northern Mexico, always been thin and nervous, but especially so during the past two years; had had no disease except bronchitis at 3, since when she has been subject to a peculiar metallic, croupy kind of cough. She always had a voracious appetite, more noticeable the last year or so, and has a marked craving for meat, particularly when undone or raw, taking a bone whenever she could find one with meat on it and gnawing at it like a dog. Her parents had never suspected a tape worm, but about six weeks before bringing her to me, after she had taken a couple of tablets of a new laxative (Fenolaxa), she passed a piece of tape worm about a foot and a half long. Her mother, who is a trained nurse, then gave her a dose of Male fern and she passed a tape worm thirty feet long by actual measurement. Her mother failed to find the head of the animal, but that was probably because she had never seen one and didn't know what to look for. A week or so later single sections of a tape worm began to appear again in the stools and the child was given a dose of "Vermifuge" but with no effect. The next day she was given pumpkin seeds, but as nothing happened she was brought to me. As she had been on a semi-starvation diet for three days I had her take one-sixth of a bottle of A's Tape Worm Remedy the next morning with instructions to follow it up with magnesium sulfate every two hours and a salt enema. One dose was given at 7 a. m. and another an hour later and the salts after another hour, but it took a second dose of salts before the entire worm finally came at 4 p. m. It was bottled up and brought to me for examination. The head was "armless," having just the four suckers of the Taenia Saginata and the total length was eighteen feet. The individual segments showed the branching of the ovaries characteristic of the beef tape worm and the diagnosis was easily established. The measurements of the segments in the middle of the animal corresponded to those given in Anders, but toward the "tail" the segments became longer and narrower and thicker so that the patient's mother who had noticed the great difference, asked me whether there were not two separate worms. Careful piecing of them together proved their continuity and showed it was but one animal. I believe, however, that the child had two worms and that the thirty-foot animal which came away with the first treatment came away entire; or can a tape worm grow eighteen feet in five weeks?

This case has demonstrated to me the value of thorough work even in such a naturally repulsive subject, as it enabled me to give a clear and definite answer to the questions of the parents. The child's improvement in health was immediate and obvious and I have added one more grateful family to the adherents of the medical profession.

Case 2. An American lady, age 40, married, four children, came to this country twenty years ago on account of pulmonary hemorrhages and tuberculosis well begun in one lung. She has had no return of her trouble since living in this dry climate, despite the fact that she spent several years in the tropics after coming here. She had a nervous breakdown eight years ago as a result of nursing sick relatives, and she suffered for two years with neuralgia of the heart,—a regular "false angina pectoris"—and later had neuralgic headaches for which she took Antikamnia three times a day during a period of four months, thereby proving that she had a strong heart. At that time she had bloating of the bowels, a symptom which nowadays always makes me think of tape worms—but as her bowels were regular and she had no other symptoms of indigestion, no attention was paid to it. The neuralgia gradually yielded to treatment and no other symptoms of importance have appeared during the past two years except a growing nervousness and weakness and a sensation as of something moving in the right iliac region. At first she thought she was pregnant, the sensation was so similar to that of "life" at three months. Afterwards when she knew she was not pregnant, she laughingly spoke of the sensation —which still persisted—as if it was an animal moving around inside of her. Latterly she felt the movements in the stomach itself, but she never seriously entertained the idea that she might have a tape worm. In fact she was horror-struck at the suggestion. Her appetite was about normal, but she was greatly bothered with bloating and a sensation of "smothering." She had been in the habit of eating rare or undone meat for years, it having been recommended to her as a good diet for a tuberculous person. In view of all these facts I advised her to watch her stools carefully for pieces of tape worm, and, sure enough, after a meal of "enchilladas" she discovered several lengths of tape worm, in all about five or six feet. She accordingly submitted herself for treatment, and after abstaining from all solid food for twenty-four hours and taking several doses of fruit salts, she took two-thirds of a bottle of "Remover" the first thing in the morning and within three-fourths of an hour passed about half of the animal. She then took the remainder of the contents of the bottle and with the aid of a strong salt water enema, succeeded in bringing away the rest of the worm, head and all. The total length was about twenty feet. It was a Taenia Saginata.

I have prescribed for four other cases of tape worm, but in none of them did I have an opportunity to make a study of the nature of the worm, so I can't include them in this report, except in so far as they contribute to the symptomatology of tape worms in general.

The most frequent symptom that I have observed is that of tympanites or "bloating." Indefinite nervous symptoms of various kinds are next in order of frequency, weakness, disturbed appetite, either excessive or not, up to normal.

Dr. Pfender states that Taenia Saginata, although without hooks, seems to be the most difficult to expel. The formula that I have used can, therefore, be given the credit for getting rid of two worms on the first attempt, and in a third case it succeeded in getting rid of a worm that had resisted several other treatments during a period of more than three years.

Apropos of the doctor's saying that no reports of deaths from either Taenia Saginata or Taenia Solium had been received in the United States of America, I would like to suggest that case No. 2 was very much afraid when she learned that she had a tape worm because she never had forgotten the case of a little girl friend who died because of being unable to get rid of one.

The formula of A's "Remover" is no secret and can be had on application.

---

To the present, the best results in the treatment of injuries of the lungs have been obtained by conservative therapy.—*American Journal of Surgery.*

